# Role of genetic polymorphisms of the *RNASEL* gene on familial prostate cancer risk in a Japanese population

**DOI:** 10.1038/sj.bjc.6601075

**Published:** 2003-08-12

**Authors:** H Nakazato, K Suzuki, H Matsui, N Ohtake, S Nakata, H Yamanaka

**Affiliations:** 1 Department of Urology, Gunma University School of Medicine, 3-39-22, Showa-machi, Maebashi, Gunma, 3718511, Japan

**Keywords:** *RNASEL* gene, familial prostate cancer, Japanese population

## Abstract

The *RNASEL* gene on chromosome 1q25 has been identified as a prostate cancer susceptibility gene. We screened for *RNASEL* germline mutations in familial prostate cancer patients, and performed a case–control study to examine the association of specific variants with prostate cancer risk in the Japanese. Three variants within the *RNASEL* gene, G282A, G1385A and T1623G were identified. G1385 and T1623G variants result in previously reported Arg462Gln and Asp541Glu variants, respectively. The novel G282A variant does not cause amino-acid substitution. A case–control study consisting of 101 familial prostate cancer cases and 105 noncancer controls showed that the Gln/Gln genotype of codon462 was observed in 7.6% of controls. However, the Gln/Gln genotype was not observed in cases, and reduced prostate cancer risk (odds ratio (OR)=0.061, *P*=0.014). The Asp/Asp genotype of codon541 increased the familial prostate cancer risk (OR=7.37, *P*=0.0004). In subset analysis, a significant association was observed in patients with more than two affected members (OR=3.15, *P*=0.028), and weak associations were found in patients with metastatic disease (OR=2.40, *P*=0.11) and high-grade disease (Gleason score ⩾7) (OR=3.07, *P*=0.14). These findings suggested that the polymorphic changes within the *RNASEL* gene may be associated with familial prostate cancer risk in a Japanese population.

Familial clustering of prostate cancer has been studied due to its clinical significance as a high-risk group of prostate cancer (Carter *et al*, 1992, 1993) and its genetic significance as a biological model to identify susceptibility genes (Berthon *et al*, 1998; Berry *et al*, 2000; Bratt, 2000, 2002). Six prostate cancer susceptibility loci (*HPC1* at 1q24-25 (Smith *et al*, 1996), *PCAP* at 1q42-43 (Berthon *et al*, 1998), *HPCX* at Xq27-28 (Xu *et al*, 1998), *CAPB* at 1p36 (Gibbs *et al*, 1999), *HPC20* at 20q13 (Berry *et al*, 2000) and *HPC2* at 17p12 (Tavtigian *et al*, 2001)) and two candidate susceptibility genes (*HPC2/ELAC2* at 17q (Tavtigian *et al*, 2001) and *RNASEL* at 1q24-25 (Carpten *et al*, 2002)) have been reported.

*RNASEL* was recently identified, and germline mutations were cosegregated within families with prostate cancer linked to the *HPC1* region, at 1q24-25 (Carpten *et al*, 2002). In Finnish patients, the truncation mutation at base pair position 793 (Glu265 → stop) was associated with hereditary prostate cancer (Rökman *et al*, 2002). Wang *et al* (2002) reported the association of the Arg462Gln genotype with familial prostate cancer risk in a United States population. *RNASEL* encodes the 2′,5′-oligoisoadenylate-synthetase-dependent ribonuclease L protein (Zhou *et al*, 1993), and is involved in the interferon-regulated 2-5A system that regulates cell proliferation and apoptosis (Hassel *et al*, 1993; Castelli *et al*, 1998).

The incidence of prostate cancer was much lower in Japan in comparison with the Western countries (Nakata *et al*, 1996). However, it has been increasing annually, and the understanding of the pathophysiology of this disease is warranted in Japan. Although, the frequency of familial/hereditary prostate cancer pedigrees is lower than that in Western countries (Ohtake *et al*, 1998; Ohtake *et al*, 2002), information on genetic characteristics of Japanese familial/hereditary prostate cancer would be interesting from the viewpoint of ethnic differences. In the present study, we analysed *RNASEL* germline mutations in familial prostate cancer in Japan, and investigated the significance of the *RNASEL* gene in genetic susceptibility in a Japanese population.

## MATERIALS AND METHODS

### Patients

The present study included 101 prostate cancer patients with a family history of prostate cancer. The numbers of affected family members were two out of 72 patients, three out of 14 patients and four out of 15 patients. In all, 29 patients with three or more affected family members were categorised into hereditary prostate cancer according to Carter's criteria (Carter *et al*, 1993). Prostate cancer was confirmed histologically at Gunma University Hospital and its affiliated hospitals. Ages ranged from 40 to 88 years with a mean age of 70.0 years. Clinical stages were A in 2, B in 41, C in 32, D in 24 and unknown in two patients according to Jewett's staging system. Gleason scores were less than 7 in 25 and equal or greater than 7 in 76 patients. Controls were recruited from outpatient clinics at Gunma University Hospital. Controls were excluded if they had an abnormal PSA level (i.e. >4.0 ng ml^−1^), and abnormal digital rectal examination, and a previous diagnosis of cancer. Ages ranged from 51 to 88 years with a mean of 71.2 years. No significant differences in age were observed between cases and controls. The Ethical Committee of Gunma University approved this study, and all participants were enrolled under informed consent.

### Single-strand conformation polymorphism (SSCP) and direct sequencing

Mutations in the coding sequence of six exons of the *RNASEL* gene in familial prostate cancer patients were screened by the SSCP method. The sequences for the PCR primers covering the coding sequence for six exons were provided by Dr Carpten (the National Human Genome Research Institute, National Institutes of Health). Genomic DNA was isolated from whole blood using a GENOMIX kit (Talent srl. Treisete, Italy). Samples were diluted to 10 *μ*g *μ*l^−1^ and stored at −20°C. PCR reactions were performed in a total 25 *μ*g reaction volume containing 20 ng of genomic DNA, primers (10 *μ*M of each forward and reverse primers), 1 × PCR buffer (1.5 mM MgCl_2_, 10 mM Tris (pH 9.0), 50 mM KCl and 0.1% Triton X-100), dNTPs (20 mM), and 1 U of AmpliTaq Gold polymerase (PE Applied Biosystems, Foster City, CA, USA). The PCR primer amplification was performed in a GeneAmp® 9700 thermalcycler (PE Applied Biosystems). Cycling conditions were 95°C for 10 min for one cycle; 94°C for 30 s, 60°C for 30 s, 72°C for 30 s for 35 cycle; followed by an elongation cycle of 72°C for 10 min. PCR products (3 *μ*l) were mixed with 7 *μ*l of denaturing solution (formamide with 10 mM NaOH), were incubated at 95°C for 5 min, and were chilled on ice immediately. Samples were electrophoresed at 6 V cm^−1^ for 15 h at 4°C in GMA™ (gene mutation analysis) Gels in 30 mM Tris-acetate EDTA (Elchrom Scientific AG, Switzerland). After electrophoresis, gels were stained with cyber-gold, and were visualised under UV irradiation. To confirm the accuracy of bands detected by the SSCP method, at least two PCR products were selected, and were sequenced using an automated ABI Prism 310 Genetic Analyzer (PE Applied Biosystems) according to the instructions of the manufacturer. To facilitate the genotyping of Arg462Gln and Asp541Glu polymorphisms of the *RNASEL* gene, we designed primers covering these polymorphic sites. The sequences of primers for Arg462Gln were 5′-cac agc ggg aag tct ctt gt-3′ (GenBank accession number NM_021133; nt 1202-1221); forward, and 5′-ggt ggg tgt atc cac agg ac-3′ (GenBank accession number NM_021133; nt 1450-1431); reverse. Those for Asp541Glu were 5′-ccttgagattcctcccatca-3′ (GenBank accession number AL138776; nt 88952-88933); forward, and 5′-ccaggatggaagagacgatg-3′ (GenBank accession number AL138776; nt 88701-88720); reverse.

### Statistical analysis

The *χ*^2^ test was performed to evaluate whether the distribution of genotype frequency of Arg462Gln and Asp541Glu polymorphisms of the *RNASEL* gene varied among cases and controls. Odds ratios (ORs) were calculated as an estimate of relative risk, and 95% confidence intervals (CIs) were calculated from unconditional logistic regression models. Genotypes were compared between cases and controls. We also performed logistic regression analysis whether clinical stage, pathological grade, age and number of affected family members were associated with RNASEL genotypes. The categories of clinical stage were localised (stages A–C) or metastatic (stage D), and that of pathological grade was Gleason score <7 or ⩾7. Ages were divided into <70 or ⩾70 years. The SPSS Professional Statistics™ (SPSS inc., Chicago, IL, USA) was used for all statistical analyses.

## RESULTS

Mutation screening of the coding sequence of six exons showed one altered band in PCR product amplified by the primer set-1a. 2 (GenBank accession number NM_021133, nt 105-419), several patterns in the primer set-1d (GenBank accession number AL138776, nt 92278-91873) and the primer set-3 (GenBank accession number AL138776, nt 88897-88565) (Table 1

**Table 1:** Analysis of the *RNASEL* gene is patients with familial prostate cancer in Japan

**Exon**	Codon	**Nucleotide change**	**Amino-acid change**
2	94	G282A	None
2	462	G1385A	Arg → Gln
4	541	G1623T	Asp → Glu

). Direct sequencing revealed the G → A transition at base pair position 282 (Figure 1

**Figure 1:**
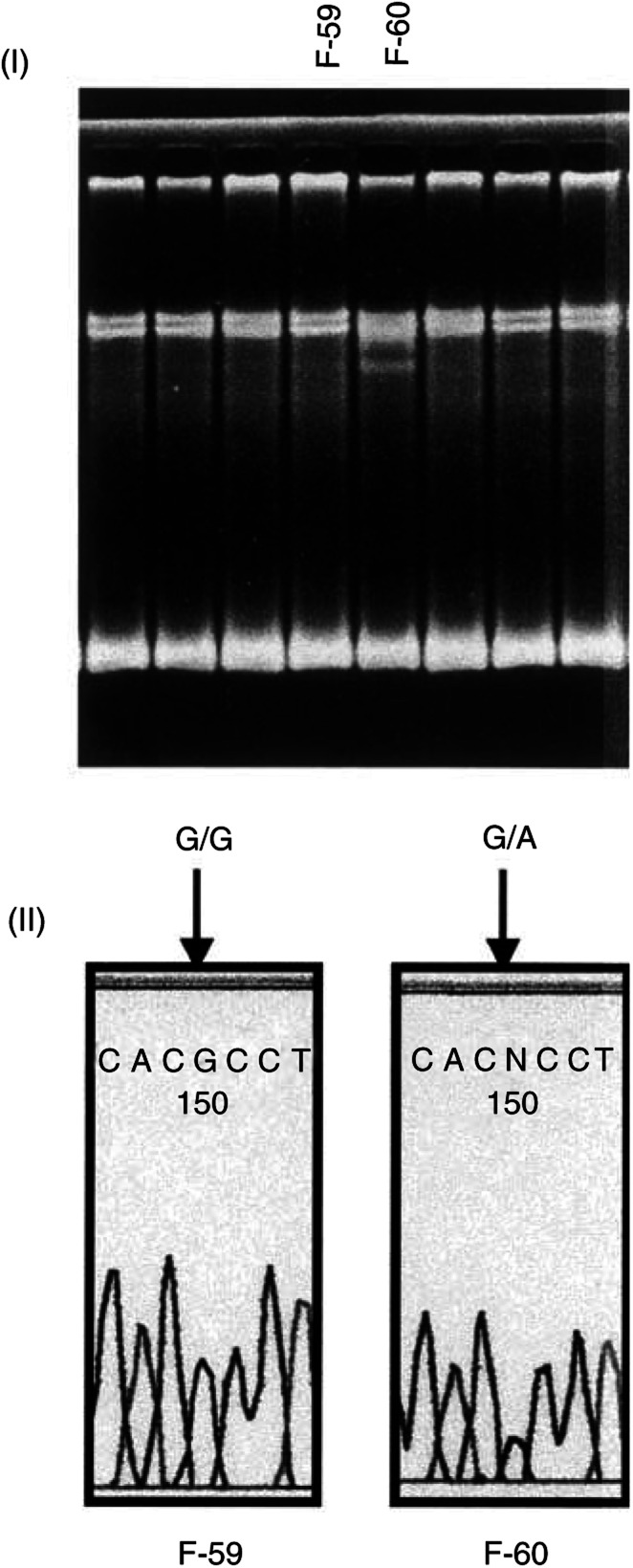
Detection of G282A mutation of the *RNASEL* gene. (I) An aberrant pattern of SSCP was observed in F-60. The pattern of F-59, the affected brother of F-60, was normal. (II) Direct sequencing showed G/A heterozygous pattern of G282A in F-60. Wild-type G/G genotype was observed in F-59.

). In this patient, this transition caused no amino-acid substitution (acg: Thr → aca:Thr). We performed three independent PCR reactions and direct sequencing and confirmed the G282A mutation. This patient was 64 years old at diagnosis of prostate cancer, and had stage B disease and a Gleason score of seven. No mutation was found in the affected brother (71 years at diagnosis, stage C, Gleason score 6) of this patient and controls at base pair position 282.

**Figure 2:**
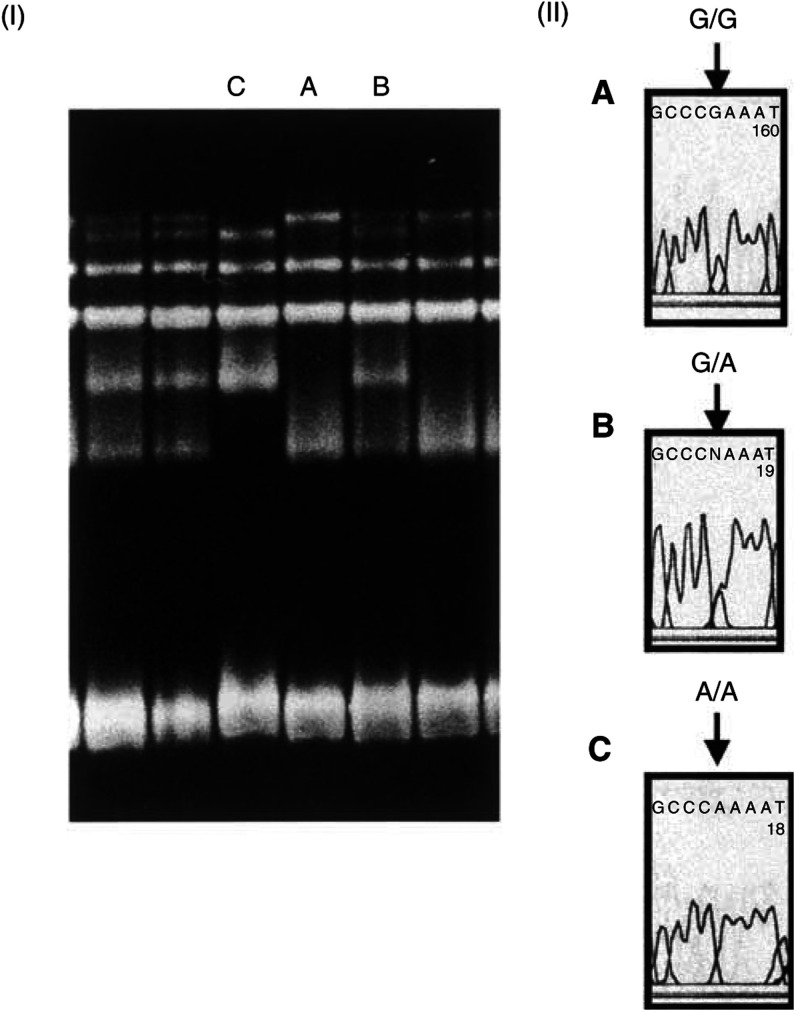
Detection of G1385A mutation of the *RNASEL* gene. (I) Three patterns were observed by SSCP analysis (**A–C**). (II) Direct sequencing showed G/G, G/A and A/A corresponding to A, B and C, respectively.

Direct sequencing of the primer set-1d and the primer set-3 showed Arg462Gln and Asp541Glu polymorphic sites, respectively (Figures 2

and 3

**Figure 3:**
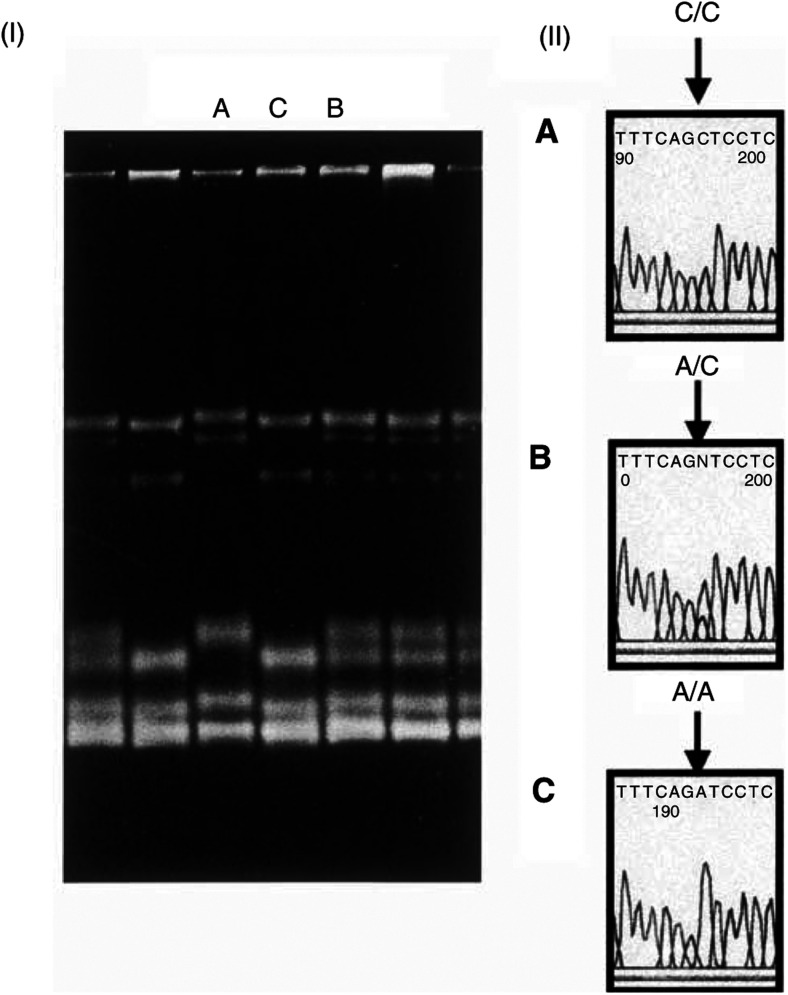
Detection of G1623 T mutation of the *RNASEL* gene. (I) Three patterns were observed in the analysis (**A–C**). (II) Direct sequencing showed C/C, C/A and A/A corresponding to **A**, **B** and **C**, respectively. Note that the sequences appearing in the figures are reverse complement due to using the reverse primer for sequencing.

). In controls, Arg/Arg, Arg/Gln and Gln/Gln genotypes were found in 71 (67.6%), 26 (24.8%) and 8 (7.6%), respectively (Table 2 Table 2

**Table 2:** Association of Arg462Gln and Asp462Gln genotypes of RNASEL with prostate cancer risk

	**No. of subjects (%)**			
**Genotype**	**Case**	**Control**	**OR (95% CI)**	***P*-value**
Arg462Gln				
Arg/Arg	69 (68.3%)	71 (67.6%)	1.0 (Ref.)	
Arg/Gln	32 (31.7%)	26 (24.8%)	1.27 (0.73–2.21)	0.45
Gln/Gln	0 (0.0%)	8 (7.6%)	0.061 (0.035–0.11)	0.014
				
Asp541Glu				
Glu/Glu	51 (50.5%)	59 (56.2%)	1.0 (Ref.)	
Glu/Asp	32 (31.7%)	43 (41.0%)	0.86 (0.49–1.50)	0.62
Asp/Asp	18 (17.8%)	3 (2.9%)	6.94 (3.98–12.10)	0.0009
				
Glu/Glu+Glu/Asp	83 (82.2%)	102 (97.1%)	1.0 (Ref.)	
Asp/Asp	18 (17.8%)	3 (2.9%)	7.37 (4.23–12.86)	0.0004

). In familial prostate cancer cases, Arg/Arg and Arg/Gln genotypes were found in 69 (68.3%) and 32 (31.7%), respectively. No Gln/Gln genotype was found in any cases, and this genotype tended to reduce prostate cancer risk (OR=0.0061, 95% CI=0.035–0.11, *P*=0.014). Regarding the Asp541Glu genotype, Glu/Glu, Glu/Asp and Asp/Asp genotypes were found in 59 (56.2%), 43 (41.0%) and three (2.9%) in controls, respectively. In cases, Glu/Glu, Glu/Asp and Asp/Asp genotypes were found in 51 (50.5%), 32 (31.7%) and 18 (17.8%), respectively. The Asp/Asp genotype increased prostate cancer risk to a significant degree (OR=6.94, 95% CI=3.98–12.10, *P*=0.0009, in comparison with the Glu/Glu genotype; OR=7.37, 95% CI=4.23–12.86, *P*=0.0004, in comparison with Glu/Glu + Glu/Asp genotypes).

Stratification of cases according to clinical stage and pathological grade was performed (Tables 3

**Table 3:** Relation between Arg462Gln and Asp541Glu genotypes and pathological grade

	**No. of subjects (%)**			
	**Gleason score**			
**Genotype**	**<7**	**⩾7**	**OR (95% CI)**	***P*-value**
Arg462Gln				
Arg/Arg	19 (76.0%)	50 (65.8%)	1.0 (Ref.)	
Arg/Gln	6 (24.0%)	26 (34.2%)	1.65 (0.94–2.87)	0.69
Gln/Gln	0 (0.0%)	0 (0.0%)		
				
Asp541Glu				
Glu/Glu	14 (56.0%)	37 (48.7%)	1.0 (Ref.)	
Glu/Asp	9 (36.0%)	23 (30.3%)	0.97 (0.55–1.69)	0.95
Asp/Asp	2 (8.0%)	16 (21.1%)	3.03 (1.74–5.28)	0.16
				
Glu/Glu+Glu/Asp	23 (92.0%)	60 (78.9%)	1.0 (Ref.)	
Asp/Asp	2 (8.0%)	16 (21.1%)	3.07 (1.76–5.35)	0.14

and 4

**Table 4:** Relation between Arg462Gln and Asp541Glu genotypes and clinical stage of prostate cancer

	**No. of subjects (%)**			
**Genotype**	**Localised**	**Metastatic**	**OR (95%CI)**	***P*-value**
Arg462Gln	51 (68.%)			
Arg/Arg	24 (32.0%)	17 (70.8%)	1.0 (Ref.)	
Arg/Gln	0 (0.0%)	7 (29.2%))	0.88 (0.50–1.53)	0.79
Gln/Gln		0 (0.0%)		
				
Asp541Glu				
Glu/Glu	39 (52.0%)	12 (50.0%)	1.0 (Ref.)	
Glu/Asp	25 (33.3%)	5 (20.8%)	0. 65 (0.37–1.13)	0.46
Asp/Asp	11 (14.7%)	7 (29.2%)	2.07 (1.19–3.61))	0.21
				
Glu/Glu+Glu/Asp	64 (85.3%)	17 (70.8%)	1.0 (Ref.)	
Asp/Asp	11 (14.7%)	7 (29.2%)	2.40 (1.37–4.18)	0.11

Two cases were excluded due to lack of staging information.

). Although no significant differences were observed in the Arg462Gln genotype in the clinical stage, the Asp/Asp genotype of codon541 tended to be frequently observed in patients with metastatic disease (OR=2.40, 95% CI=1.37–4.18, *P*=0.11, in comparison with Glu/Glu + Glu/Asp genotypes) (Table 3). As for pathological grade, no significant tendency was observed in the Arg462Gln genotype, however, the Asp/Asp genotype of codon541 tended to be frequently seen in patients with high-grade prostate cancer (OR=3.07, 95% CI=1.76–5.35, *P*=0.14, in reference with Glu/Glu+Glu/Asp genotypes) (Table 4). Stratification of subjects according to age showed a similar tendency of distribution of both the genotypes as demonstrated in Table 4 (data not shown). The frequency of the Asp/Asp genotype of codon541 was significantly higher in families with more than two affected members compared with that in families with two affected members (OR=3.15, 95% CI=1.81–5.49, *P*=0.028, in comparison with Glu/Glu+Glu/Asp genotypes) (Table 5

**Table 5:** Relation between Arg462Gln and Asp541Glu gennotypes and the number of affected family members

	**No. of subjects (%)**			
	**No. of affected members**			
**Genotype**	**<2**	**>2**	**OR (95% CI)**	***P*-value**
Arg462Gln				
Arg/Arg	49 (68.1%)	20 (69.0%)	1.0 (Ref.)	
Arg/Gln	23 (31 9%)	9 (31.0%)	0.96 (0.55–1.67)	0.93
Gln/Gln	0 (0.0%)	0 (0.0%)		
				
Asp541Glu				
Glu/Glu	37 (51.4%)	14 (48.3%)	1.0 (Ref.)	
Glu/Asp	26 (36.1%)	6 (20.7%)	0.61 (0.35–1.06)	0.37
Asp/Asp	9 (12.5%)	9 (31.0%)	2.64 (1.52–4.61)	0.08
				
Glu/Glu+Glu/Asp	63 (87.5%)	20 (69.0%)	1.0 (Ref.)	
Asp/Asp	9 (12.5%)	9 (31.0%)	3.15 (1.81–5.49)	0.028

).

## DISCUSSION

In the present study, we identified three variants within the *RNASEL* gene among 101 patients with familial prostate cancer. Two of them were previously reported variants, Arg461Gln and Asp541Glu. We found one novel mutation of G–A transition at codon94. This mutation does not cause amino-acid substitution, and was not observed in the affected brother of the case. Truncating mutation E265X was reported to cosegregate in high-risk US families with hereditary prostate cancer (Carpten *et al*, 2002). E265X was also associated with hereditary prostate cancer in Finnish patients (Rökman *et al*, 2002). In the present study, E265X mutation was not observed in cases and controls. Rennert *et al* (2002) found a novel founder mutation, 471delAAAG, in Ashkenazi Jews, and reported that this mutation was associated with prostate cancer risk in Ashkenazi men. In the present study, this mutation was not observed in patients with familial prostate cancer in Japan.

The most significant finding in the present study was the association of the Asp541Glu genotype with prostate cancer risk. In the Japanese population, the frequency of the wild-type Asp/Asp genotype was rare (2.9% in controls), and the Glu541 variants were prevalent in controls. We calculated ORs of Glu/Asp and Asp/Asp genotypes in comparison with the Glu/Glu genotype as shown in Table 2. The Asp/Asp genotype was significantly associated with familial prostate cancer risk (*P*=0.0004), with OR=7.37. Furthermore, subset analysis showed that this significant association was observed with more than two affected family members, that is, hereditary prostate cancer (OR=3.15, *P*=0.028), and a weak association was observed with metastatic stage disease (OR=2.40, *P*=0.11) and high-grade (Gleason score ⩾7) (OR=3.07, *P*=0.14). These associations have not been reported by any other studies (Casey *et al*, 2002; Rökman *et al*, 2002; Wang *et al*, 2002), and might be a specific feature of familial prostate cancer in Japan.

Another significant finding was the low frequency of the Gln462 variant in patients with familial prostate cancer. In the present study, we did not find the Gln/Gln genotype in patients with familial prostate cancer. The present findings supported the results reported by Wang *et al* (2002). They observed that the low frequency of the Gln 462 genotype was significantly associated with familial prostate cancer. Subset analysis showed that this association was observed in the younger group, localised stage group and low-grade disease. However, we did not find any significant differences in the frequencies of Arg/Arg and Arg/Gln genotypes in subset analysis. On the other hand, Rökman *et al* (2002) reported a higher, but not significant frequency, of the Gln462 variant. Recently, Casey *et al* (2002) reported that the Gln462 variant has three times less enzymatic activity than the wild type. They found that the Gln462 variant is significantly associated with prostate cancer risk. Controls in Casey's study were nonaffected brothers, however, those in other studies including the present study were population-based controls (Rökman *et al*, 2002 ; Wang *et al*, 2002). However, the exact reasons for discrepancies between these results are unknown. Further studies will be necessary to confirm the significance of the Arg462Gln genotype in the risk of familial prostate cancer.

Limitations of the present study are the relatively small sample number and the inclusion criteria of familial prostate cancer. We started to collect familial prostate cancer pedigrees in 1994 (Ohtake *et al*, 1998), and the frequency of familial prostate cancer was about 3% (Ohtake *et al*, 2002). The incidence of prostate cancer in Japan is markedly lower than those in Western countries (Nakata *et al*, 1996), and the national awareness of prostate cancer has increased only in these two decades. These situations have made it difficult to diagnose prostate cancer at an early age, to identify affected members in equal or more than three generations and three members in one family. Due to these reasons, we enrolled prostate cancer patients from at least two affected members in families. The present study design used did not involve prospective recruitment of study subjects into a study cohort. This point is another limitation of the present study, and it could cause several biases.

In summary, we found three variants within the *RNASEL* gene, G282A, G1385A and T1623G. The G1385 and G1623 T variants result in previously reported Arg462Gln and Asp541Glu variants, respectively. The G282A variant does not cause amino acid substitution, and this mutation was identified for the first time. A case–control study showed that the Gln/Gln genotype of codon462 was not observed in cases, and reduced prostate cancer risk to a significant degree. The Asp541Glu genotype had a marked association with familial prostate cancer risk. The Asp/Asp genotype increased the familial prostate cancer risk, and was associated with risk of patients with many affected family members. These associations need further large-scale and prospective studies to confirm the consequences of the *RNASEL* gene on familial prostate cancer.
